# Detection of Marburg Virus Disease in Guinea

**DOI:** 10.1056/NEJMc2120183

**Published:** 2022-06-30

**Authors:** Fara R. Koundouno, Sophie Duraffour, N’Faly Magassouba, A complete list of authors is available with the full text of this letter at NEJM.org., Fara R. Koundouno, Liana E. Kafetzopoulou, Martin Faye, Annick Renevey, Barré Soropogui, Kékoura Ifono, Emily V. Nelson, Aly A. Kamano, Charles Tolno, Giuditta Annibaldis, Saa L. Millimono, Jacob Camara, Karifa Kourouma, Ahmadou Doré, Tamba E. Millimouno, Fernand M.B. Tolno, Julia Hinzmann, Hugo Soubrier, Mette Hinrichs, Anke Thielebein, Glaucia Herzer, Meike Pahlmann, Georges A. Ki-Zerbo, Pierre Formenty, Anaïs Legand, Michael R. Wiley, Ousmane Faye, Moussa M. Diagne, Amadou A. Sall, Philippe Lemey, Aïssatou Bah, Stephan Günther, Sophie Duraffour, Sakoba Keita, N’Faly Magassouba

**Affiliations:** Bernhard Nocht Institute for Tropical Medicine (BNITM) Hamburg, Germany; Bernhard Nocht Institute for Tropical Medicine (BNITM) Hamburg, Germany; Laboratoire des Fièvres Hémorragiques Virales en Guinée (LFHVG) Conakry, Guinea; Bernhard Nocht Institute for Tropical Medicine (BNITM) Hamburg, Germany; KU Leuven Leuven, Belgium; Institut Pasteur Dakar Dakar, Senegal; Bernhard Nocht Institute for Tropical Medicine (BNITM) Hamburg, Germany; Université Gamal Abdel Nasser Conakry, Guinea; Bernhard Nocht Institute for Tropical Medicine (BNITM) Hamburg, Germany; World Health Organization (WHO) Guinea Conakry, Guinea; Médecins Sans Frontières (MSF) Belgium Conakry, Guinea; Bernhard Nocht Institute for Tropical Medicine (BNITM) Hamburg, Germany; Bernhard Nocht Institute for Tropical Medicine (BNITM) Hamburg, Germany; Université Gamal Abdel Nasser Conakry, Guinea; Bernhard Nocht Institute for Tropical Medicine (BNITM) Hamburg, Germany; Université Gamal Abdel Nasser Conakry, Guinea; Bernhard Nocht Institute for Tropical Medicine (BNITM) Hamburg, Germany; Bernhard Nocht Institute for Tropical Medicine (BNITM) Hamburg, Germany; Bernhard Nocht Institute for Tropical Medicine (BNITM) Hamburg, Germany; Bernhard Nocht Institute for Tropical Medicine (BNITM) Hamburg, Germany; Bernhard Nocht Institute for Tropical Medicine (BNITM) Hamburg, Germany; Bernhard Nocht Institute for Tropical Medicine (BNITM) Hamburg, Germany; Bernhard Nocht Institute for Tropical Medicine (BNITM) Hamburg, Germany; Bernhard Nocht Institute for Tropical Medicine (BNITM) Hamburg, Germany; World Health Organization (WHO) Guinea Conakry, Guinea; World Health Organization (WHO) Geneva, Switzerland; World Health Organization (WHO) Geneva, Switzerland; University of Nebraska Medical Center Omaha, NE; Institut Pasteur Dakar Dakar, Senegal; Institut Pasteur Dakar Dakar, Senegal; Institut Pasteur Dakar Dakar, Senegal; KU Leuven Leuven, Belgium; Université Gamal Abdel Nasser Conakry, Guinea; Bernhard Nocht Institute for Tropical Medicine (BNITM) Hamburg, Germany; Agence Nationale de Sécurité Sanitaire Conakry, Guinea; Bernhard Nocht Institute for Tropical Medicine (BNITM) Hamburg, Germany; Université Gamal Abdel Nasser Conakry, Guinea

On August 2^nd^, 2021, a patient from Temessadou M’Boké town (Gueckédou prefecture) died with presentation of hemorrhage from several natural orifices (Figure 1A and Supplementary Appendix, available with the full text of this letter at NEJM.org). On August 3^rd^, the initial diagnosis of Marburg virus (MARV) was made on a post-mortem buccal swab sample by real time reverse transcription PCR with a cycle threshold (Ct) value of 13.4. Field investigation teams were deployed and validation of the diagnostic finding in two additional laboratories occurred within a few days (Figure 1A). In-country metagenomic next generation sequencing allowed for full-length MARV genome recovery (99.3%) and phylogenetic analysis indicated that the new Guinea MARV strain clusters with MARV strains isolated from bats in Sierra Leone and from humans in Angola (Figure 1B and Supplementary Appendix). Close monitoring for a period of 21 days identified that all contacts remained asymptomatic, and no additional cases were detected.

Forest Guinea, along with other areas of West Africa including Sierra Leone, is predicted to be environmentally suitable for zoonotic transmission of MVD by bats and particularly by *Rousettus aegyptiacus* (ERB) which has been identified as a natural MARV reservoir host.^1–3^ Most MARV bat reservoir hosts are present in the forested region of Guinea, particularly in Koundou, which is close to the case emergence location (Figure1A and Supplementary Appendix). The patient had limited social interactions and lived in a household of four people. There was no evidence of travel history to country outside Guinea for the patient or his close contacts, nor contacts to returning travelers. He was a farmer living in close contact with nature and wildlife and may therefore have experienced repeated exposure to an environment or food contaminated with excreta of MARV-infected bats. Community surveys showed that while he may have harvested wild fruits for personal consumption, there was no suggestion of visits to caves, nor hunting activities for bushmeat, including bats, or its consumption. Traditional practices of bushmeat consumption or preparation (i.e., direct exposure to bodily fluids) cannot be fully excluded as they would not be disclosed in the context of the national ban that was enforced following the 2021 EVD outbreak.

The new Guinea MARV and the Angola MARV clade share a common ancestor that probably existed in 1965 [95% confidence interval: 1944,1981, Bayesian molecular clock analysis]. This indicates that about 55 years ago, these lineages diverged from a common ancestor, and each evolved independently in its respective reservoir host with the Guinea MARV remaining undetected until this 2021 spillover event. This time scale of decades provided ample opportunity for the virus to be dispersed over large distances by bat migration. One could draw a parallel with the emergence of the West-African Ebola virus lineage (Makona) that diverged from a central African ancestor and independently evolved in its host until the spillover event happened.^4^ In the case of MARV, the basal clustering of bat MARV in Sierra Leone suggests that even the Angola outbreak may have had its eventual roots in West Africa (Figure 1B).

Both the epidemiology and phylogenetic history argue against the possibility the new MVD may be imported. Overall, it seems plausible that the MVD emergence in Guinea is due to a zoonotic transmission event from a bat reservoir at the end of July 2021.

The isolated living habit of the patient likely played a role in minimizing the risk of secondary infections. Notably, capacity building programs, long-term collaborative partnerships, and establishment of decentralized laboratory capacities with well-trained staff have been valuable for a timely laboratory diagnosis. These same capacities proved key during the recent reemergence of EVD in Guinea.^5^

## Supplementary Material

Supplement

## Figures and Tables

**Figure 1:**
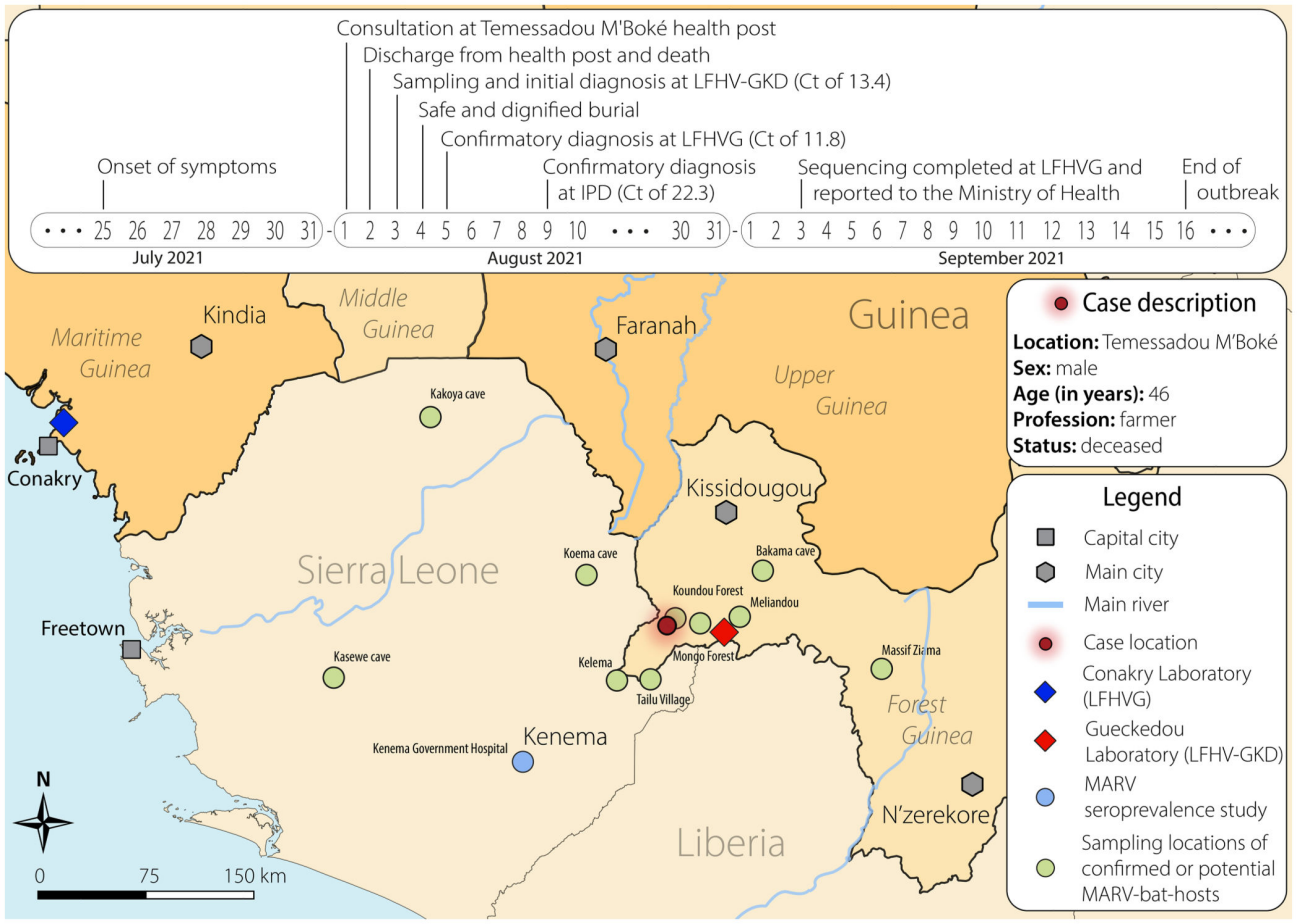

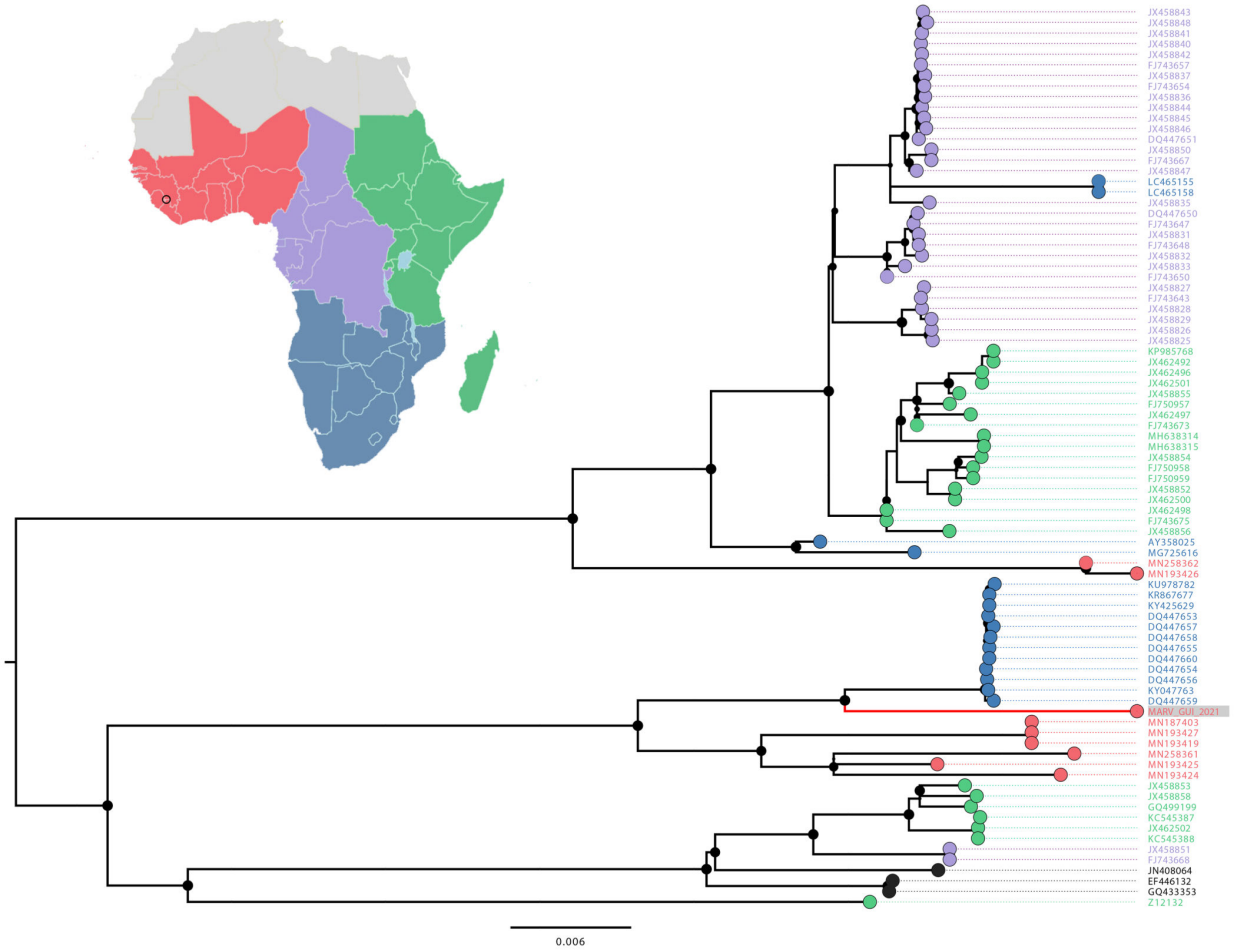
Map and timeline of events of the case occurrence (A) and phylogenetic analysis of MARV genomes (B) (A) The top panel highlights the timeline of events while the map depicts parts of Guinea, Sierra Leone and Liberia with a focus on Forest Guinea and the Gueckédou prefecture where the MVD emerged. Note that on 3 September, LFHVG further shared the Marburg virus sequence with the public (https://virological.org/t/marburg-virus-sequence-from-guinea-2021/755) to support the public health response as well as the development and evaluation of Marburg virus disease diagnostics and therapeutics. The map depicts locations of the laboratory based in Gueckédou (LFHV-GKD), that of Conakry (LFHVG), and other relevant locations of sites that have reported (i) evidence of MARV circulation in bats^1^ and in humans^3^ in Sierra Leone, and (ii) identification of bat species known to be potential reservoir hosts of MARV in Guinea, including Méliandou, the location of the 2014-2016 Ebola virus disease outbreak, as well as Mongo Forest, Koundou Forest, Bakama cave, and Massif Ziama. The names and GPS locations of study sites from published works were used (Supplementary Appendix). The map was drawn using QGIS software version 3.22.0 and Adobe Illustrator^®^. Ct, cycle threshold; LFHV-GKD, “Laboratoire des Fièvres Hémorragiques Virales de Gueckédou”; LFHVG, “Laboratoire des Fièvres Hémorragiques Virales de la Guinée” ; IPD, “Institut Pasteur de Dakar”. (B) The map highlights four geographic regions of the African Union with known reports of MARV circulation (red, West Africa; green, East Africa; purple, Central Africa; dark blue, Southern Africa). This same color code is applied in the phylogenetic tree as per geographic origin of the sequence. Note that sequences in black refer to MARV cases that occurred outside Africa. The new Guinea MARV genome (MARV_GUI_2021, red and grey background, long branch) clusters between the Sierra Leone bat MARV clade (red) and the human Angola clade (blue). The internal node shapes are proportional to bootstrap support and accession numbers are provided. The accession numbers of the new MARV Guinea are OK665848 for LFHVG and OL702894 for IPD.
