# Supplementary material for: Detection of Marburg Virus Disease in Guinea
Source: N Engl J Med. Author manuscript; Available in PMC 2022 Dec 19. (PMC7613962; doi:10.1056/NEJMc2120183)
Supplement: Supplement [file EMS155619-supplement-Supplement.pdf]

Supplementary Appendix

Detection of Marburg Virus Disease in Guinea

List of investigators: Fara R. Koundouno, M.Sc.[1a], Liana E. Kafetzopoulou, Ph.D.[2a], Martin Faye, Ph.D.[3], Annick Renevey, Ph.D.[1], Barré Soropogui, M.Sc.[4], Kékoura Ifono, B.Sc.[1], Emily V. Nelson, Ph.D.[1], Aly Antoine Kamano, M.P.H., M.D.[5], Charles Tolno, M.P.H., M.D.[6], Giuditta Annibaldis, Ph.D.[1], Saa L. Millimono, B.Sc.[1], Jacob Camara, Pharm.D.[4], Karifa Kourouma, B.Sc.[1], Ahmadou Doré, B.Sc.[4], Tamba Elie Millimouno, B.Sc.[1], Fernand M'Bemba Tolno, B.Sc.[1], Julia Hinzmann, M.L.T.[1], Hugo Soubrier, M.Sc.[1], Mette Hinrichs, M.L.T[1], Anke Thielebein, Ph.D.[1], Glaucia Herzer, M.Sc.[1], Meike Pahlmann, Ph.D.[1], Georges A. Ki-Zerbo, M.D.[5], Pierre Formenty, D.V.M.[7], Anaïs Legand, M.P.H.[7], Michael R. Wiley, Ph.D.[8], Ousmane Faye, Ph.D.[3], Moussa Moïse Diagne, Ph.D.[3], Amadou Alpha Sall, Ph.D.[3], Philippe Lemey, PhD.[2], Aïssatou Bah, B.Sc.[4], Stephan Günther, M.D., PhD.[1], Sakoba Keita, M.D.[9], Sophie Duraffour, Ph.D.[1b], N'Faly Magassouba, Ph.D. [4b].

[a] Equal contribution first authors
[b] Equal contribution last authors

[1]Bernhard Nocht Institute for Tropical Medicine (BNITM), Hamburg, Germany; [2]KU Leuven, Leuven, Belgium; [3]Institut Pasteur Dakar, Dakar, Senegal; [4]Laboratoire des Fièvres Hémorragiques Virales en Guinée (LFHVG), Université Gamal Abdel Nasser, Conakry, Guinea; [5]World Health Organization (WHO) Guinea, Conakry, Guinea; [6]Médecins Sans Frontières (MSF) Belgium, Conakry, Guinea; [7]World Health Organization (WHO), Geneva, Switzerland; [8]University of Nebraska Medical Center, Omaha, NE, USA; [9]Agence Nationale de Sécurité Sanitaire, Conakry, Guinea

Table of Contents

Methods 2
- Case description 2
- Geographic description of the area and community interviews about the case 2
- Contact tracing, monitoring and alerts 3
- Laboratories involved 3
- Laboratory diagnosis and viral RNA extraction 4
- Metagenomic sequencing 4
- Phylogenetic analysis and divergence time estimation 6
- Genome analysis 6
- Hypothesis for virus emergence 7

Tables 8
- Table S1 8

References 9

Acknowledgements 10

Methods

Case description

On 1 August 2021, a 46 years-old male farmer from Temessadou M'Boké center, a town localized in the sub-prefecture Koundou Lengo Bengou, prefecture of Gueckédou, Guinea, came for a consultation at the health post of Temessadou M'Boké. His symptoms, which began on 25 July, included fever (body temperature of 38 °C), headache, general malaise, cold extremities, nausea, anorexia, gingivorrhagia, pallor, and vertigo. The patient was hypotensive with blood pressure of 60 over 40 mmHg. A malaria rapid diagnostic test was positive and a diagnosis of malaria accompanied with severe anemia was made prompting treatment with antimalarial medication (Artemether/Lumefantrine or Coartem®), paracetamol, iron with supplements (Zincofer®), anti-parasitic and anti-worm medicines (Mebendazole®), vitamin B complex, and rehydration salts. On 2 August, the physician suggested the patient to be transferred to a larger health care center of Koundou Lengo Bengou but the family declined, and the patient returned home. He died later that day in his residence with a presentation of hemorrhages from several natural orifices including mouth, nose and ears. The patient was sampled post-mortem (buccal swab); no blood sample was collected.

Geographic description of the area and community interviews about the case

The case was living in the western part of the Gueckédou prefecture which shares a border with Sierra Leone. This area is mainly composed of degraded forest however it still contains few forested zones (e.g., Mongo Forest of Temessadou town, and Koundou Forest of the sub-prefecture Koundou Lengo Bengou). It was reported that the case had prior history of mental illness and limited social interactions, and lived in a household of 4 people, including himself, his wife, their child and his mother. Together with his wife, he was mainly occupied during the day with farming

activities around Temessadou M'Boké town, thus living in close contact with nature and wildlife. He was also known to wander both short and long distances from his residence during the day and would only return to town at night. There was no travel history of the patient or his close contacts to countries outside Guinea. There were no records of cave visits. While he may have harvested wild fruits for personal consumption, there was however no suggestion of hunting activities for bushmeat (e.g., bats or monkeys) or its consumption. Following community interviews, there was no evidence of monkeys living in the neighboring forests (i.e., Mongo Forest and Koundou Forest) in recent years. Villagers also did not recall any unusual observations of increased animal death rates in either domestic or wild animal population in the last year. Bats are naturally observed in the region of the patient but no further investigations of species identification or colony sizes estimates were performed as part of this work.

Contact tracing, monitoring and alerts

Epidemiological surveys led to the identification of 173 contacts, of which 14 considered as high-risk including close family members and the treating physician. All contacts remained asymptomatic during the 21 days of monitoring and none of them were sampled. Along the active surveillance period, none of the suspected cases (i.e., with a case definition matching that of MARV or viral hemorrhagic fevers) were found positive for MARV.

Laboratories involved

The three laboratories involved in the laboratory analysis were LFHV-GKD or "Laboratoire des Fièvres Hémorragiques Virales de Gueckédou" located in Gueckédou, LFHVG or "Laboratoire des Fièvres Hémorragiques Virales de la Guinée" located in Conakry and IPD or "Institut Pasteur de Dakar" from Senegal. LFHV-GKD performed the initial diagnosis; LFHVG and IPD performed confirmatory diagnosis and sequencing.

Laboratory diagnosis and viral RNA extraction

In the two laboratories in Guinea, the buccal swab sample was tested for the presence of EBOV and MARV by RT-PCR. The dry swab was eluted in 0.8 ml of nuclease-free water. Viral RNA was extracted from 140 µl of sample using the QIAmp viral RNA kit (Qiagen, Germany). Initial diagnosis at LFHV-GKD was made using the commercially available real-time RT-PCR assay, RealStar® Filovirus Screen RT-PCR Kit 1.0 (altona Diagnostics, Germany; referred as 'Filovirus screen') on the Rotor-Gene platform (Qiagen) according to the manufacturer's instructions. Confirmatory diagnosis at LFHVG was made using the same RT-PCR kit as described above. In addition, typing of filovirus species was performed using the RealStar® Filovirus Type RT-PCR Kit 1.0 (altona Diagnostics, Germany; referred as 'Filovirus Type'). Differential diagnosis at LFHV-GKD was made with the same RNA extract using other commercial assays from altona Diagnostics including the RealStar® Lassa Virus RT-PCR Kit 2.0, RealStar® Yellow Fever Virus RT-PCR Kit 1.0, and RealStar® SARS-CoV-2 RT-PCR Kit 1.0. The sample was tested and found negative for other viruses including EBOV, Lassa virus, Yellow fever virus and SARS-CoV-2.

At IPD, viral RNA was extracted from 140 µl of swab sample using the QIAamp Viral RNA Mini Kit (Qiagen) according to the manufacturer's instructions and eluted in nuclease-free water. Extracted RNA was tested using a Taqman based real time quantitative RT-PCR assay with specific Marburg virus primers targeting the nucleoprotein as previously described.[1]

Metagenomic sequencing

Metagenomic sequencing was independently performed at two laboratories, LFHVG and IPD.

At LFHVG, extraction and metagenomic library preparation for next generation sequencing (NGS) on MinION platform (Oxford Nanpore Technologies, United Kingdom) were performed as described previously.[2] Briefly, extracted viral RNA from 120 µl of buccal swab was used for

sequencing. RNA was treated with DNase (TURBO DNase, Thermo Fisher Scientific), randomly reverse-transcribed, and amplified using a Sequence Independent Single Primer Amplification (SISPA) approach as in.[2] MinION sequencing library was prepared using SQK-LSK109 and following manufacturer's instructions (Oxford Nanopore Technologies). Sequencing libraries were sequenced for ~48hr using R9.4.1 Flow Cells on the Mk1C device (Oxford Nanopore Technologies) using MinKNOW version 21.02.2. Majority consensus sequence was obtained with bases called at a minimum depth of 20 x and support fraction of 70%. Any base location that did not fulfil the depth and support fraction criteria was assigned an "N" as per IUPAC ambiguity notation. The complete sequence has been submitted to GenBank under the accession number OK665848.

At IPD, metagenomic sequencing was performed as previously described.[3] Briefly, viral RNA was extracted from 140 µl of swab sample. The viral RNA was prepared and enriched using the Illumina RNA Prep with enrichment, the (L) Tagmentation kit (Illumina, San Diego, CA, USA), according to the manufacturer's recommendations, and a pan viral probe panel that included MARV specific targets. The Illumina MiSeq platform using the Miseq reagents kit v3 (Illumina, San Diego) was used. Reads were quality trimmed by Prinseq-lite and consensus MARV genome sequence was generated using an in-house de novo genome assembly pipeline in the Empowering the Development of Genomics Expertise (EDGE) platform (*https://edgebioinformatics.org/)*. The complete sequence is available in GenBank under the accession number OL702894.

The two MARV full length sequences obtained independently by LFHVG and IPD are identical on overlapping regions.

Phylogenetic analysis and divergence time estimation

All MARV sequences with known sampling times and a length > 400 bp were downloaded from the NIAID Virus Pathogen Database and Analysis Resource (ViPR). Only unique sequences were kept and the genomes for the Ozolin (accession number: AY358025) and Musoke (Z12132) strain were additionally added. Five short sequences that appeared to be outliers in a regression analysis of root-to-tip divergence against sampling time using TempEst were removed.[2] Phylogenetic reconstruction was performed using IQTree.[3] The general time-reversible (GTR) model was specified with gamma-distributed rate variation and performed ultrafast bootstrapping[4] with 1000 replicates to obtain node support values. For divergence time estimation using BEAST,[3] the complete genomes from the phylogenetic data set were used while the sequences for the Ravn virus, a close relative of MARV, were removed. As part of our Bayesian model, the same GTR+gamma model as for the standard phylogenetic inference, an uncorrelated relaxed clock model and a flexible Bayesian skygrid model was specified.[3] Markov Monte Carlo chain analyses was run for 50,000,000 generations and ensured sufficiently high effective sampling sizes for all continuous parameters in our model.

Genome analysis

To better visualize the nucleotide and amino acid differences of the new MARV genome as compared to other MARV sequences, a pairwise comparison with the MARV reference sequence (RefSeq) from Kenya (NC_001608), as well as with a prototype sequence representative of the Angola clade (DQ447654) was made (**Table S1**). The overall genetic diversity was of 7.6% with the East African MARV whereas it was only of 2.4% with the Angola MARV. Percent differences in nucleotide range from 4.7% (Minor nucleoprotein VP30) to 8.9% (Glycoprotein GP) and in amino acids from 0.7% (Matrix protein VP40) to 7.9% (Glycoprotein GP) between the new

MARV as compared to the RefSeq MARV. When comparing the new MARV with a prototype sequence of its closest clade, the Angola MARV, the percent differences in nucleotide range from 1.4% (Minor nucleoprotein VP30) to 2.1% (Matrix protein VP24 and Glycoprotein GP) and in amino acids from 0% (Minor nucleoprotein VP30) to 2.8% (Glycoprotein GP). The glycoprotein coding sequence harbored the highest nucleotide and amino acid variability as compared to all other genes. The percent variations observed among gene products of different MARV clades (East African MARV versus Angola MARV) are in line with previous observations.[5]

Hypothesis for virus emergence

The hypothesis for MARV emergence in Guinea is supported by the presence of bat reservoir hosts in the country. Insectivorous and fruit bats from Guinea are known to carry Ebolavirus,[6] including Bombali virus found in *Mops condylurus* bats,[7] as well as a broad range of other human pathogens.[8,9] ERB and other bat species including *Epomops franqueti*, *Hypsignathus monstrosus*, *Miniopterus inflatus*, *Rhinolophus eloquens* and *Hipposideros spp* have been identified as potential MARV reservoir hosts following serological and or PCR findings.[10,11] Most of these bat species are present in the forested region of Guinea, particularly in Koundou which is close to the identified MVD case.[6,8,10,12] The locations of sites and names of sites from published works that have reported evidence of MARV circulation in bats in Sierra Leone[10] and identified bat species in Guinea known to be potential reservoir hosts of MARV[6,8,12] have been used to annotate **Figure 1A**. In addition, while serological data about MARV exposure in humans in Guinea is still lacking, its neighboring country Sierra Leone depicts MARV seroprevalence in humans up to 10.7%.[13] This reinforces the assumption of undetected MARV exposure in Guinea.

Tables

Table S1

Pairwise comparison of the nucleotide and amino acid content of the new MARV genome with the MARV reference sequence from GenBank or with a prototype sequence representative of the Angola clade.

Brief description	Pairwise comparison with Marburg marburgvirus reference sequence*		Pairwise comparison with Marburg marburgvirus sequence from Angola#	
**Complete genome**				
Length in nucleotides in reference sequence*#	19,111		19,114	
Length in nucleotides in query§	19,058		19,058	
Percent query coverage (%)	99.7		99.7	
Identities$	17,650		18,658	
**Percent identity (%)**	**92.4**		**97.6**	
**Percent difference (%)**	**7.6**		**2.4**	
	**Coding sequence**	**Protein**	**Coding sequence**	**Protein**
**Nucleoprotein NP**				
Length in nucleotides or amino acids	2,088	695	2,088	695
Percent query coverage (%)	100	100	100	100
Identities$	1,961	677	2,056	686
**Percent identity (%)**	**93.9**	**97.4**	**98.5**	**98.7**
**Percent difference (%)**	**6.1**	**2.6**	**1.5**	**1.3**
**Polymerase complex protein VP35**				
Length in nucleotides or amino acids	990	329	990	329
Percent query coverage (%)	100	100	100	100
Identities$	941	325	974	328
**Percent identity (%)**	**95.1**	**98.8**	**98.4**	**99.7**
**Percent difference (%)**	**4.9**	**1.2**	**1.6**	**0.3**
**Matrix protein VP40**				
Length in nucleotides or amino acids	912	303	912	303
Percent query coverage (%)	100	100	100	100
Identities$	861	301	896	300
**Percent identity (%)**	**94.4**	**99.3**	**98.2**	**99.0**
**Percent difference (%)**	**5.6**	**0.7**	**1.8**	**1.0**
**Glycoprotein GP**				
Length in nucleotides or amino acids	2,046	681	2,046	681
Percent query coverage (%)	100	100	100	100
Identities$	1,881	627	2,003	662
**Percent identity (%)**	**91.9**	**92.1**	**97.9**	**97.2**
**Percent difference (%)**	**8.1**	**7.9**	**2.1**	**2.8**
**Minor nucleoprotein VP30**				
Length in nucleotides or amino acids	846	281	846	281
Percent query coverage (%)	100	100	100	100
Identities$	806	276	834	281
**Percent identity (%)**	**95.3**	**98.2**	**98.6**	**100.0**
**Percent difference (%)**	**4.7**	**1.8**	**1.4**	**0.0**
**Matrix protein VP24**				
Length in nucleotides or amino acids	762	253	762	253
Percent query coverage (%)	100	100	100	100
Identities$	720	250	746	252
**Percent identity (%)**	**94.5**	**98.8**	**97.9**	**99.6**
**Percent difference (%)**	**5.5**	**1.2**	**2.1**	**0.4**
**RNA-dependent RNA polymerase L**				
Length in nucleotides or amino acids	6,996	2,331	6,996	2,331
Percent query coverage (%)	100	100	100	100

Identities$	6,599	2,245	6,880	2,309
**Percent identity (%)**	**94.3**	**96.3**	**98.3**	**99.1**
**Percent difference (%)**	**5.7**	**3.7**	**1.7**	**0.9**

*The reference sequence or RefSeq with GenBank accession NC_001608 was used; it is designated as Marburg marburgvirus isolate Marburg virus/H.sapiens-tc/KEN/1980/Mt. Elgon-Musoke

#The MARV sequence with GenBank accession DQ447654 was used as prototype sequence representative of the Angola clade (Figure 1B); it is designated as Lake Victoria marburgvirus - Angola2005 strain Ang1381.

§The new MARV sequence with GenBank accession OL702894 was used here as query in all pairwise comparisons. Note that the MARV sequence obtained with MinION technology (GenBank accession OK665848) is identical to OL702894 on called nucleotides and has a nucleotide length of 18,972 (excluding ambiguous bases) yielding 99.3% coverage as compared to NC_001608 and DQ447654.

$Number of identical nucleotides or amino acids at the same position in the alignment.

References

1. Weidmann M, Muhlberger E, Hufert FT. Rapid detection protocol for filoviruses. J Clin Virol 2004;30:94-9.
2. Kafetzopoulou LE, Pullan ST, Lemey P, et al. Metagenomic sequencing at the epicenter of the Nigeria 2018 Lassa fever outbreak. Science 2019;363:74-7.
3. Keita AK, Koundouno FR, Faye M, et al. Resurgence of Ebola virus in 2021 in Guinea suggests a new paradigm for outbreaks. Nature 2021.
4. Minh BQ, Nguyen MA, von Haeseler A. Ultrafast approximation for phylogenetic bootstrap. Mol Biol Evol 2013;30:1188-95.
5. Towner JS, Khristova ML, Sealy TK, et al. Marburgvirus genomics and association with a large hemorrhagic fever outbreak in Angola. J Virol 2006;80:6497-516.
6. De Nys HM, Kingebeni PM, Keita AK, et al. Survey of Ebola Viruses in Frugivorous and Insectivorous Bats in Guinea, Cameroon, and the Democratic Republic of the Congo, 2015-2017. Emerg Infect Dis 2018;24:2228-40.
7. Karan LS, Makenov MT, Korneev MG, et al. Bombali Virus in Mops condylurus Bats, Guinea. Emerg Infect Dis 2019;25.
8. Hermida Lorenzo RJ, Cadar D, Koundouno FR, et al. Metagenomic Snapshots of Viral Components in Guinean Bats. Microorganisms 2021;9.
9. Lacroix A, Vidal N, Keita AK, et al. Wide Diversity of Coronaviruses in Frugivorous and Insectivorous Bat Species: A Pilot Study in Guinea, West Africa. Viruses 2020;12.
10. Amman BR, Bird BH, Bakarr IA, et al. Isolation of Angola-like Marburg virus from Egyptian rousette bats from West Africa. Nat Commun 2020;11:510.
11. Olival KJ, Hayman DT. Filoviruses in bats: current knowledge and future directions. Viruses 2014;6:1759-88.
12. Mari Saez A, Weiss S, Nowak K, et al. Investigating the zoonotic origin of the West African Ebola epidemic. EMBO Mol Med 2015;7:17-23.
13. O'Hearn AE, Voorhees MA, Fetterer DP, et al. Serosurveillance of viral pathogens circulating in West Africa. Virol J 2016;13:163.

Acknowledgements

The BNITM acknowledges technical support from Steven T. Pullan, Ph.D., Public Health England.
